# The Relationship between Serum Bilirubin and Inflammatory Bowel Disease

**DOI:** 10.1155/2019/5256460

**Published:** 2019-04-15

**Authors:** Xiaojing Zhao, Linzhen Li, Xueting Li, Jiajia Li, Di Wang, Hongjie Zhang

**Affiliations:** Department of Gastroenterology, First Affiliated Hospital of Nanjing Medical University, Nanjing, Jiangsu Province 210029, China

## Abstract

The associations between serum total bilirubin (sTB) levels, inflammatory marker levels, and disease activity are not well understood in patients with inflammatory bowel disease (IBD). The present study investigated the association between sTB levels and disease activity in patients with IBD. We conducted a retrospective study with a total of 242 consecutive patients with Crohn's disease (CD) and 211 consecutive patients with ulcerative colitis (UC). The Crohn's Disease Activity Index (CDAI) score was used to assess disease activity in patients with CD and the Mayo score of patients with UC. 255 clinically healthy subjects comprised the control group, which come from the same geographic area as the IBD group. We retrieved the clinical and laboratory parameters of patients with IBD from the medical records. Patients with IBD displayed significantly lower sTB levels than controls. sTB levels were negatively associated with C-reactive protein (CRP), erythrocyte sedimentation rate (ESR), fecal calprotectin (FC), and hemoglobin (Hb) levels in patients with IBD. Additionally, there was a negative association between sTB levels and the CDAI score of patients with CD. sTB levels were also negatively associated with the Mayo score of patients with UC. IBD patients had lower sTB levels when compared with controls, and there was a negative correlation between sTB levels and disease activity in patients with IBD. Increased reactive oxygen species production in IBD is likely to be responsible for increased consumption of bilirubin in patients with IBD, leading to further intestinal injury. Reducing oxidative stress may be therapeutic for these patients.

## 1. Introduction

Inflammatory bowel disease (IBD), comprised of ulcerative colitis (UC) and Crohn's disease (CD), is a group of relapsing chronic inflammatory diseases involving the digestive tract. The pathogenesis of IBD is complex and remains undetermined, being thought to result from an interaction between susceptibility genes, intestinal flora disorders, environmental factors, and an excessive immune response [1–4]. Chronic colonic inflammation is associated with increased oxidative stress and disruption of the intestinal homeostasis [5, 6]. Thus, it is necessary to determine the potential mechanism of increased oxidative stress in patients with IBD [7].

Several previous studies have found reduced bilirubin levels in IBD [8–10]. Bilirubin is one of the most potent endogenous antioxidants, which plays an important role in lipid peroxidation prevention [11]. Increased reactive oxygen species production in IBD is likely to be responsible for the increased consumption of bilirubin in patients with IBD, leading to intestinal injury [8–10]. Schieffer et al. [10] conducted a retrospective case-control study and found that patients with UC exhibited lower levels of serum total bilirubin (sTB) than healthy controls. However, the relationship between sTB levels and the clinical stage of UC has not been established yet. Leníček et al. [8] found lower sTB levels in patients with CD and determined that they were likely due to increased oxidative stress mediated by inflammation rather than genetic predisposition. However, they did not correlate sTB levels with levels of laboratory inflammatory parameters such as fecal calprotectin (FC), erythrocyte sedimentation rate (ESR), or C-reactive protein (CRP). Bilirubin is the main product of the heme catabolism pathway in the general circulation [12]. However, previous studies did not correlate sTB with hemoglobin (Hb) levels.

The levels of sTB in Chinese patients with UC have previously been evaluated. However, the relationship between sTB and IBD activity has not been established yet; the association between sTB levels and CRP, ESR, FC, and Hb levels is not well understood in Chinese patients with IBD. We hypothesized that patients with IBD would demonstrate lower sTB levels than healthy controls and that there would be a negative correlation with disease activity. To verify this hypothesis, a retrospective study of patients with IBD was conducted on patients in the Department of Gastroenterology of the First Affiliated Hospital of Nanjing Medical University.

## 2. Materials and Methods

### 2.1. Ethical Considerations

The study protocol was approved by the First Affiliated Hospital of Nanjing Medical University, Nanjing, China. The Institutional Review Board and Ethics Committee approved the protocol. All participants have signed the informed consent in this study.

### 2.2. Subjects

In this retrospective study, a total of 242 consecutive patients with CD and 211 consecutive patients with UC were evaluated between January 2014 and October 2018 in the Department of Gastroenterology of the First Affiliated Hospital of Nanjing Medical University. The IBD diagnosis was made in accordance with the European Crohn's and Colitis Organisation (ECCO) guidelines [13, 14]. In addition, we used the Crohn's Disease Activity Index (CDAI) to evaluate the disease activity of patients with CD and the Mayo score to assess patients with UC [13, 14]. Patients with Gilbert syndrome and primary sclerosing cholangitis or any other autoimmune disease, which might influence sTB levels, were excluded from the study. The normal control group (*n* = 255) comprised clinically healthy subjects, which come from the same geographic area as the IBD group.

The association between sTB levels and disease activity (CDAI and Mayo scores) was determined in all patients with IBD for whom bilirubin data were available. To eliminate the potential confounding effects of abnormal liver function on sTB levels mediated by secondary factors (i.e., underlying liver disease or concomitant drug treatment), subjects with any abnormal level of the liver function tests (defined as alanine aminotransferase (ALT) > 50.0 U/L, aspartate aminotransferase (AST) > 40.0 U/L, alkaline phosphatase > 120.0 U/L, and gamma-glutamyl transferase > 60.0 U/L) were not included in this study. We retrieved the clinical and laboratory parameters from the medical record systems while sTB levels were measured.

### 2.3. Statistical Analysis

GraphPad Prism 5 (GraphPad Software, USA) and SPSS version 21.0 (IBM SPSS Statistics, USA) were used for statistical analysis in the study. Normally distributed variables are expressed as mean ± standard deviation (SD), and nonnormally distributed variables are expressed as median (interquartile range). Numbers and percentages are used to represent categorical variables. One-way analysis of variance (ANOVA) followed by Tukey's post hoc test was utilized for normally distributed variables among three or more groups. The Mann-Whitney rank-sum test was employed for comparisons of nonnormally distributed variables. Correlations were determined using Pearson's or Spearman's coefficient, as appropriate, to determine the association between bilirubin and the various clinical and laboratory parameters. We assessed the relationships between clinical and laboratory parameters with the CDAI and Mayo scores using univariate analysis. A *P* value < 0.05 was set as statistically significant.

## 3. Results

### 3.1. Basic Clinical Features of IBD Patients

The basic clinical features of patients with IBD are illustrated in Table 1. This retrospective case-control study included 242 patients with CD (average age = 31.69 years, sex ratio (male/female) = 169/73), 211 patients with UC (average age = 47.05 years, sex ratio (male/female) = 124/87), and 255 controls (average age = 47.36 years, sex ratio (male/female) = 134/121).

### 3.2. sTB in Patients with IBD

**Figure 1:**
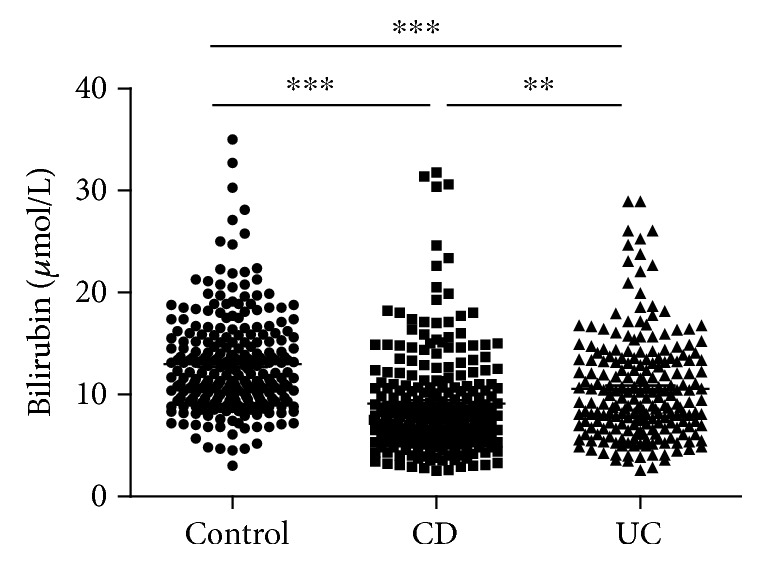
Serum bilirubin levels in CD patients (*n* = 242), UC patients (*n* = 211), and healthy controls (*n* = 255). ^∗∗^*P* < 0.01; ^∗∗∗^*P* < 0.001.

sTB levels were significantly lower in patients with CD and UC (Figure 1) when compared with controls. Gender stratification revealed that this decrease was more prominent in females than in males (Table 2). When stratified according to age, the decrease in sTB levels was more pronounced in young patients (<30 years old, Table 2).

Analysis of sTB levels according to the disease location in patients with CD revealed a trend towards lower sTB levels in L2 (Table 3). When stratified according to the extent of disease in UC, the decrease in sTB levels was more pronounced in patients with UC with a more extensive disease pattern (E3) (Table 3).

### 3.3. Association of Clinical Parameters with sTB

**Figure 2:**
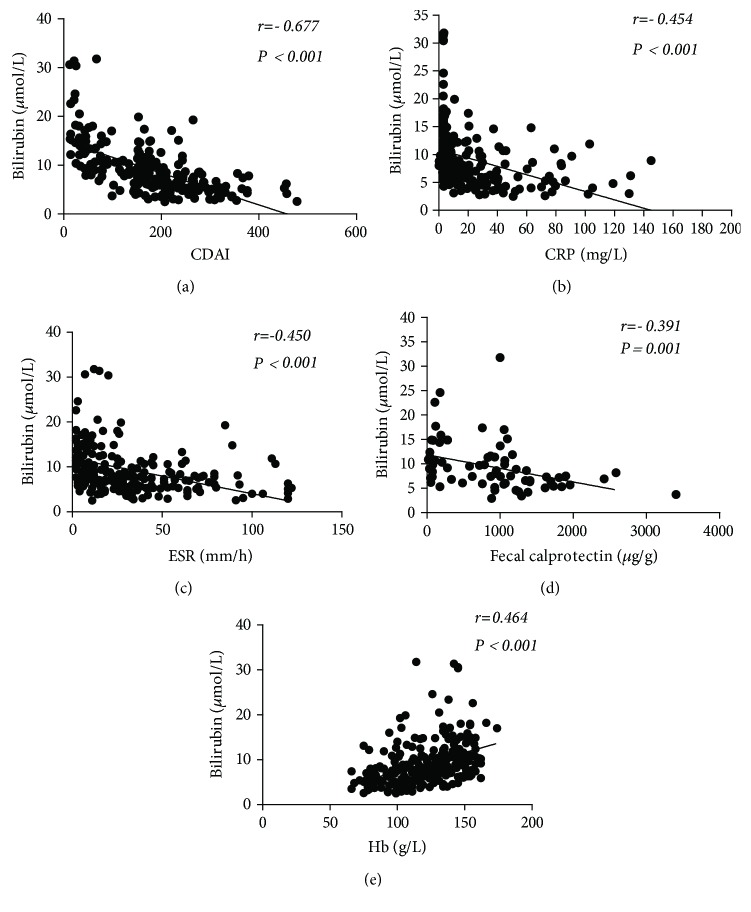
The correlation between serum bilirubin levels and (a) CDAI score (*n* = 224), (b) CRP level (*n* = 224), (c) ESR (*n* = 213), (d) FC (*n* = 70), and (e) Hb level (*n* = 242) in CD patients by using a scatter plot.

**Figure 3:**
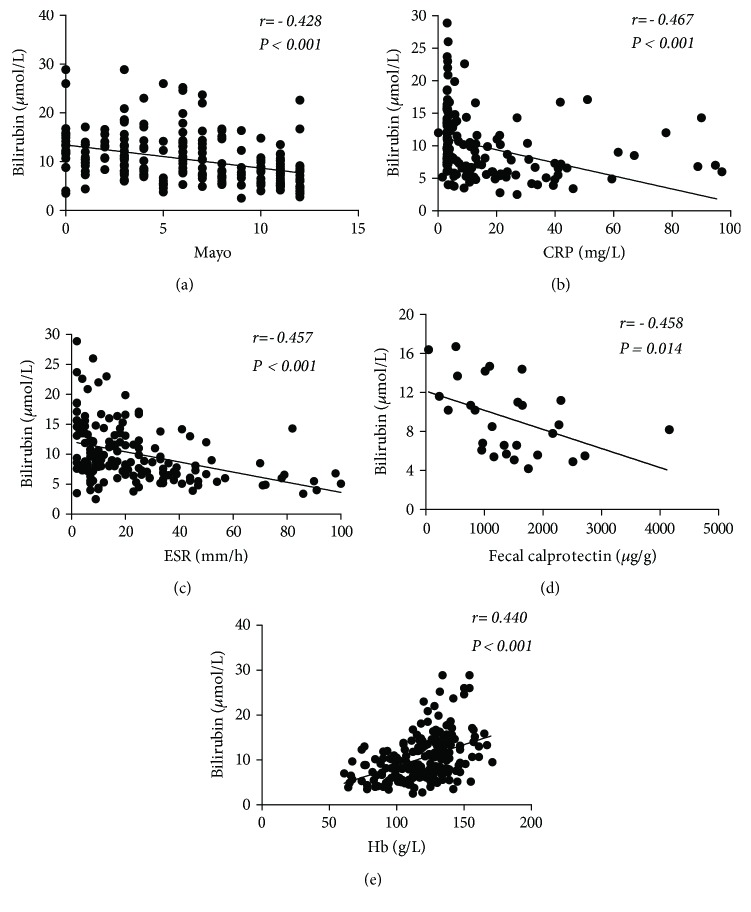
The correlation between the serum bilirubin level and (a) Mayo score (*n* = 211), (b) CRP level (*n* = 162), (c) ESR (*n* = 155), (d) FC (*n* = 28), and (e) Hb level (*n* = 210) in UC patients by using a scatter plot.

sTB levels were negatively associated with CDAI in patients with CD. Similarly, there was a negative association between sTB levels and the Mayo score of patients with UC (Figures 2(a) and 3(a)). sTB levels were found to be negatively correlated with CRP, ESR, FC, and Hb levels in patients with IBD (Figures 2 and 3(b)–3(e)).

## 4. Discussion

To summarize, we investigated the alterations in sTB levels in patients with IBD in our study. The results determined lower sTB levels in patients with IBD when compared with controls, and the sTB levels were negatively correlated with the CDAI and Mayo scores.

sTB is an end product of heme catabolism in the intravascular compartment and a common laboratory index in conventional biochemical detection. The sTB level has been considered to be a meaningful laboratory marker for the diagnosis of hepatobiliary and hemolytic disease [15, 16]. Recently, several studies demonstrated the association between sTB levels and migraines, diabetes, and chronic kidney disease [17–19]. Lower sTB levels have also been found to increase the risk of cardiovascular diseases (CVD) [20, 21]. In the present study, we detected lower sTB and Hb levels in patients with IBD; the levels were negatively correlated with the CDAI and Mayo scores of patients with CD and UC, respectively. The exact mechanism of the association between sTB levels and disease activity has not been thoroughly investigated in patients with IBD. Leníček et al. demonstrated that low sTB levels in patients with CD were a consequence of increased oxidative stress mediated by inflammation rather than genetic predisposition [8]. One possibility is that the sTB levels are influenced by abnormal immune responses directly or serum Hb levels indirectly in patients with IBD. Mounting evidence shows that sTB plays an important role in immunology [22]. Moreover, bilirubin is a potent ligand of the aryl hydrocarbon receptor, which is a member of the basic helix-loop-helix superfamily of ligand-inducible transcription factors [23, 24] and a critical regulator of regulatory T cell differentiation [25]. Additionally, serum bilirubin has been confirmed to be an effective antioxidant for hydrogen peroxide [26]. Recently, a study has revealed that sTB levels are negatively associated with disease activity in patients with rheumatoid arthritis and diabetes [27, 28]. Inflammatory conditions and immune responses are related to disease severity in patients with IBD, and higher CDAI and Mayo scores tend to indicate more severe inflammatory reactions [29, 30]. Taking these results together, it seems likely that increased inflammatory responses tend to decrease sTB levels in patients with IBD with higher CDAI and Mayo scores. This increase in inflammatory and immune responses in the intestine may also lead to a decrease in serum Hb levels and consequently cause a reduction in sTB levels in patients with IBD with severe disease activity.

There are several limitations to this study. First, our sample size is small; second, the effect of the UGT1A1 gene on the bilirubin level in patients with IBD was not examined. We also did not analyze genetic factors due to the limitations of our retrospective study. Third, sTB levels might be affected by drugs, a possibility which we did not examine. Finally, we did not determine the anti-inflammatory medication effect on sTB levels in patients with IBD. However, the present study showed that sTB levels were lower both in patients with CD and in patients with UC when compared with controls and that sTB levels negatively correlated with disease activity in patients with IBD.

## 5. Conclusion

In conclusion, results from this study confirm that Chinese patients with IBD have significantly lower levels of sTB and these are associated with IBD manifestations. Specifically, our study further demonstrated that patients with IBD with lower levels of sTB also have lower levels of Hb. Based on these data, we raise the question whether medical therapy focused on the suppression of oxidative stress may be useful for IBD. Nevertheless, these findings still need to be supported by more clinical evidence assessing biomarkers of oxidative stress in patients with IBD.

## Figures and Tables

**Table 1:** Basic clinical features of IBD patients.

	Control (*n* = 255)	IBD (*n* = 453)	
		CD (*n* = 242)	UC (*n* = 211)
Sex (male/female)	134/121	169/73^###^	124/87
Age (years)	47.36 ± 13.47	31.69±12.32^∗∗∗^	47.05 ± 15.91
Disease duration (years)	—	3.30 ± 3.69	3.99 ± 5.09

Compared with the control: ^###^*P* < 0.001; compared with the control and UC: ^∗∗∗^*P* < 0.001.

**(a):** 

Groups	Control (*n* = 255)		CD (*n* = 242)		UC (*n* = 211)	
	Serum bilirubin (*μ*mol/L)	*P*	Serum bilirubin (*μ*mol/L)	*P*	Serum bilirubin (*μ*mol/L)	*P*
Sex						
Male	13.2 (9.9-16.2) (*n* = 134)	**0.009**	8.5 (6.2-11.3) (*n* = 169)	**0.027**	9.5 (7.1-13.7) (*n* = 124)	0.339
Female	11.2 (8.9-14.4) (*n* = 121)		7.1 (5.3-9.4) (*n* = 73)		9.6 (6.6-12.8) (*n* = 87)	
Age group (yr)						
<30	11.1 (8.7-13.6) (*n* = 26)	0.266	7.8 (5.4-10.7) (*n* = 133)	0.500	7.3 (5.9-9.7) (*n* = 37)	**0.008**
30–50	12.4 (9.6-15.2) (*n* = 130)		8.0 (5.9-11.7) (*n* = 86)		9.6 (7.8-14.2) (*n* = 83)	
>50	12.3 (10.1-15.9) (*n* = 99)		7.6 (7.1-9.3) (*n* = 23)		10.8 (7.1-13.5) (*n* = 91)	

**(b):** 

Groups	Control (*n* = 255)	CD (*n* = 242)	UC (*n* = 211)	*P*
	Serum bilirubin (*μ*mol/L)	Serum bilirubin (*μ*mol/L)	Serum bilirubin (*μ*mol/L)	
Sex				
Male	13.2 (9.9-16.2) (*n* = 134)	8.5 (6.2-11.3) (*n* = 169)	9.5 (7.1-13.7) (*n* = 124)	**0.001**
Female	11.2 (8.9-14.4) (*n* = 121)	7.1 (5.3-9.4) (*n* = 73)	9.6 (6.6-12.8) (*n* = 87)	**0.001**
Age group (yr)				
<30	11.1 (8.7-13.6) (*n* = 26)	7.8 (5.4-10.7) (*n* = 133)	7.3 (5.9-9.7) (*n* = 37)	**0.002**
30–50	12.4 (9.6-15.2) (*n* = 130)	8.0 (5.9-11.7) (*n* = 86)	9.6 (7.8-14.2) (*n* = 83)	**0.001**
>50	12.3 (10.1-15.9) (*n* = 99)	7.6 (7.1-9.3) (*n* = 23)	10.8 (7.1-13.5) (*n* = 91)	**0.001**

**Table 3:** Relationship between phenotype characteristics and serum bilirubin levels of patients with IBD.

	*n* (%)	Serum bilirubin (*μ*mol/L)	*P*
*CD (n* = 242)			
Age at diagnosis (yr)			
A1 (≤16)	16 (6.6%)	6.5 (4.2-12.5)	0.453
A2 (17-40)	180 (74.4%)	8.1 (5.5-11.2)	
A3 (>40)	46 (19.0%)	7.7 (6.7-10.3)	
Location of the disease			
L1	80 (33.1%)	8.8 (6.4-12.6)	**0.023**
L2	11 (4.5%)	6.5 (5.3-9.1)	
L3	151 (62.4%)	7.4 (5.3-10.6)	
L4	28 (11.6%)	8.0 (5.8-9.7)	
Disease behavior			
B1	139 (57.4%)	8.5 (5.8-12.4)	0.047
B2	97 (40.1%)	7.5 (6.1-10.0)	
B3	6 (2.5%)	5.1 (4.5-6.7)	
P	69 (28.5%)	8.7 (6.2-13.5)	
*UC (n* = 211)			
Disease extension			
E1	41 (19.4%)	12.8 (8.9-15.1)	**0.001**
E2	57 (27.0%)	10.7 (8.1-14.3)	
E3	113 (53.6%)	8.1 (5.6-11.6)	

## Data Availability

The data used to support the findings of this study are available from the corresponding author upon request.
